# Characteristics and outcomes of patients treated with tigecycline for MDR gram-negative infections: a retrospective cohort study

**DOI:** 10.3389/fcimb.2026.1790441

**Published:** 2026-04-01

**Authors:** Shuroug A. Alowais, Atheer Aldairem, Khalid bin Saleh, Yazed S. Alsowaida, Muhannad Musaad Alharbi, Haytham A. Wali, Thamer A. Almangour

**Affiliations:** 1 Department of Pharmacy Practice, College of Pharmacy, King Saud bin Abdulaziz University for Health Sciences, Riyadh, Saudi Arabia; 2 King Abdullah International Medical Research Center, Riyadh, Saudi Arabia; 3 Ministry of the National Guard - Health Affairs, Riyadh, Saudi Arabia; 4 Department of Clinical Pharmacy, College of Pharmacy, University of Ha’il, Ha’il, Saudi Arabia; 5 Department of Pharmacy Practice, College of Clinical Pharmacy, King Faisal University, Al-Ahsa, Saudi Arabia; 6 Department of Clinical Pharmacy, College of Pharmacy, King Saud University, Riyadh, Saudi Arabia

**Keywords:** antimicrobial resistance, clinical outcomes, mortality, multidrug-resistant gram-negative bacteria, tigecycline

## Abstract

**Introduction:**

Multidrug-resistant (MDR) gram-negative pathogens pose a major therapeutic challenge, particularly in settings with limited antimicrobial options. Tigecycline is frequently used as salvage therapy. However, evidence regarding the optimal tigecycline regimen, whether used as monotherapy or in combination, remains uncertain. This study aimed to describe the characteristics, clinical, and microbiological outcomes of these cases.

**Methods:**

Retrospective, single-center study of adult hospitalized patients treated with tigecycline for MDR *Acinetobacter baumannii*, extended-spectrum β-lactamase (ESBL)-producing *Escherichia coli*, and ESBL- or Carbapenem-Resistant Enterobacterales *Klebsiella pneumoniae*. Patients were categorized into tigecycline monotherapy or combination therapy groups. The primary outcomes were clinical success and 30-day mortality. Multivariable logistic regression was used to identify predictors of outcomes. A sensitivity analysis excluding bloodstream and urinary tract infections was performed due to pharmacokinetic limitations of tigecycline.

**Results:**

Two hundred eighty were included; 116 (41.4%) received tigecycline monotherapy and 164 (58.6%) received combination therapy. Patients receiving combination therapy had higher illness severity and higher intensive care unit admissions. Overall clinical success was achieved in 53.2% of patients with a higher rate in the monotherapy group (64.7%, *p* = 0.001), and 30-day mortality was 26.1%. After adjustment, tigecycline monotherapy was not independently associated with clinical success or mortality. Sensitivity analyses excluding bloodstream and urinary tract infections showed consistent results.

**Conclusion:**

In this real-world cohort of severely ill patients with MDR gram-negative infections, no outcome advantage was observed with tigecycline combination therapy after adjustment. Clinical outcomes were primarily driven by illness severity and infection characteristics rather than treatment strategy.

## 1 Introduction

Antimicrobial resistance (AMR) is a major global threat to healthcare systems, as it is associated with increased morbidity and mortality. Moreover, infections caused by multidrug-resistant (MDR) organisms significantly increase healthcare costs due to longer hospital stays. According to the Centers for Disease Control and Prevention (CDC), an MDR organism is defined as one that shows non-susceptibility to at least one agent in three or more antimicrobial categories (Centers for Disease Control and Prevention (CDC); Magiorakos et al., 2012). In 2019, around 33,000 deaths in the U.S. were caused by infections from MDR organisms (De Rosa et al., 2015).

The burden of MDR Gram-negative pathogens is particularly significant in the Arabian Peninsula. A systematic review of 382 studies reported that the Kingdom of Saudi Arabia (KSA) had among the highest numbers of MDR isolates in the region, including 10,972 isolates of *Escherichia coli* and 3,787 isolates of *Acinetobacter baumannii*. *A. baumannii* was frequently the most prevalent MDR bacterium in KSA (Borgio et al., 2021). More recent surveillance from multiple Riyadh hospitals similarly demonstrated high rates of MDR organisms, with 89.9% of Acinetobacter spp. and approximately 60% of Klebsiella spp. and E. coli isolates identified as ESBL or carbapenemase producers (Alsaab et al., 2024). These organisms have been associated with numerous reported outbreaks and mortality rates ranging from 11% to 40% (Zowawi et al., 2013). Also, the 2019 Global Research on Antimicrobial Resistance (GRAM) Project reported that 2,500 deaths in Saudi Arabia were directly attributed to AMR. Further, 9,100 deaths occurred in which AMR was a contributing factor (The burden of antimicrobial resistance (AMR) in Saudi Arabia). The alarming trend highlights the urgent need develop new antimicrobial agents and optimize the use of existing therapies.

Among the most clinically challenging MDR pathogens are *Klebsiella pneumoniae*, *Escherichia coli*, and *Acinetobacter baumannii*, all classified within the ESKAPEE group of organisms (*Enterococcus faecium, Staphylococcus aureus, Klebsiella pneumoniae, Acinetobacter baumannii, Pseudomonas aeruginosa, Enterobacter* spp.*, and Escherichia coli*) (Hubeny et al., 2022), known for causing healthcare-associated infections and for their capacity to develop resistance through diverse mechanisms (Lolans et al., 2006). *A. baumannii* is considered a difficult-to-treat pathogen due to its ability to develop resistance through multiple mechanisms, including beta-lactamase production, permeability changes, target site modification, efflux pumps, and aminoglycoside-modifying enzymes. These mechanisms have led to strains that are pan-resistant (Centers for Disease Control and Prevention and Division of Healthcare-associated infections; Peleg et al., 2007; Lee et al., 2017).

*K. pneumoniae* and *E. coli*, also pose major clinical challenges due to the global spread of carbapenem-resistant Enterobacterales (CRE) and extended-spectrum β-lactamase (ESBL) producers. Unfortunately, carbapenem-resistant *K. pneumoniae* has spread worldwide (Woodworth et al., 2018). Among the various types of carbapenemases, *K. pneumoniae* producing Klebsiella pneumoniae carbapenemase is the most commonly identified organism in the United States (Woodworth et al., 2018). In the Gulf Cooperation Council (GCC) States, it has been found that carbapenem-resistant Enterobacteriaceae harbor carbapenemase-encoding genes of the bla_OXA-48_-type and bla_NDM-1_ (Zowawi et al., 2014). CRE not only inactivates carbapenems but also beta-lactams and cephalosporins (Ben-Ami et al., 2009). Another critical resistance mechanism is the production of ESBL, which confers resistance to beta-lactam antibiotics, including aztreonam, cephalosporins, and penicillins. These infections can be acquired in both hospital and community settings (Ben-Ami et al., 2009).

Tigecycline, a glycylcycline antibiotic, exhibits broad-spectrum activity against many MDR organisms, including ESBL producers, CRE *K. pneumoniae*, and MDR *A. baumannii*. It inhibits bacterial protein synthesis by binding to the 30S ribosomal subunit and is generally considered bacteriostatic with a long post-antibiotic effect (Yaghoubi et al., 2022). It is approved by the U.S. Food and Drug Administration (FDA) for the treatment of complicated intra-abdominal infections, community-acquired pneumonia, and complicated skin and skin structure infections using a regimen of a 100 mg loading dose, followed by 50 mg every 12 hours (Tigecycline for Injection, 50 mg/vial). However, due to concerns about low serum concentrations and increased mortality in certain populations, the FDA has issued a boxed warning advising that tigecycline should only be used when alternative treatments are not suitable. A meta-analysis of phase 3 and 4 clinical trials found a 0.6% increase in all-cause mortality among patients treated with tigecycline compared to other agents, particularly in cases of hospital-acquired and ventilator-associated pneumonia (Food and Drug Administration, 2010). Despite these concerns, tigecycline continues to be used for difficult-to-treat infections, particularly when other antibiotics are ineffective or contraindicated. Its pharmacokinetic profile enables the achievement of therapeutic concentrations by penetrating body fluids and tissues, including the liver, kidneys, heart, lungs, skin, bone, and bladder (Tombs, 1999; Stein et al., 2011; Cai et al., 2016). The ratio of the area under the concentration-time curve to the minimal inhibitory concentration (AUC/MIC) for serum tigecycline concentrations is considered a significant predictor of clinical response (Meagher et al., 2007; Passarell et al., 2008).

Although tigecycline is increasingly used as a salvage therapy for MDR Gram-negative infections in Saudi Arabia, local data describing patient characteristics, infection types, treatment patterns, and clinical outcomes remain limited. This gap is particularly important given the high regional prevalence of MDR pathogens and the ongoing reliance on tigecycline in severe or refractory infections. Therefore, this retrospective cohort study aims to describe the characteristics and clinical outcomes of patients treated with tigecycline for infections caused by MDR gram-negative organisms, including ESBL-producing *E. coli*, MDR *A. baumannii*, CRE *K. pneumoniae*, and ESBL-producing *K. pneumoniae* at a tertiary care center in Riyadh, Saudi Arabia.

## 2 Methods

### 2.1 Study design and settings

This single-center retrospective cohort study was conducted at King Abdulaziz Medical City (KAMC), a tertiary care center in Riyadh, Saudi Arabia. The study included adult inpatients treated with tigecycline for (MDR) gram-negative organisms. Organism identification and antimicrobial susceptibility testing were performed in the hospital microbiology laboratory in accordance with CLSI guidelines.

### 2.2 Patient selection and study outcomes

Eligible patients were ≥18 years of age and received tigecycline for a documented infection due to an MDR organism, including ESBL-producing *E. coli*, MDR *A. baumannii*, and CRE or ESBL-producing *K. pneumoniae*. Patients were excluded if tigecycline was prescribed for non–gram-negative infections, if the duration of therapy was <72 hours, or if outcome data were incomplete or the patient was transferred before therapy completion. Patients were categorized into two groups based on the antimicrobial regimen they received. The monotherapy group included patients treated with tigecycline alone for the targeted MDR pathogen. The combination therapy group included patients who received tigecycline plus at least one additional systemic antimicrobial agent with *in-vitro* activity against the isolate, with at least 48 hours of overlapping therapy.

The primary outcome was to describe the clinical characteristics and outcomes of patients treated with tigecycline, either as monotherapy or combination therapy, for MDR gram-negative infections. The secondary outcomes include length of hospitalization, 30-day mortality, ICU admission, and escalation of antimicrobial therapy following initial tigecycline treatment.

### 2.3 Ethical approval

This study received Institutional Review Board (IRB) approval from King Abdullah International Medical Research Center (KAIMRC) (approval number: SP23R/152/06). The study was conducted in accordance with the Declaration of Helsinki (1964) and its subsequent amendments. Informed consent was waived by the IRB.

### 2.4 Definitions

Time to tigecycline initiation: defined as the number of days from the date of the index MDR gram-negative culture to the first dose of tigecycline.

Early tigecycline start: referred to therapy initiated within 48 hours of index culture collection.

Combination therapy: defined as tigecycline administered concurrently with another active systemic antimicrobial agent for at least 48 hours of overlap.

30-day mortality: defined as death from any cause within 30 days of tigecycline initiation.

Hospital length of stay: calculated from the date of admission to discharge.

Treatment escalation: defined as the addition of new systemic antibiotics due to inadequate clinical response.

Clinical success: was defined as the absence of antibiotic escalation during tigecycline therapy and survival at 30 days post-culture.

Microbiologic failure: was defined as the persistence or regrowth of the same pathogen within 21 days or documented evidence of acquired resistance.

### 2.5 Statistical analysis

Continuous variables were assessed for normality using the Shapiro–Wilk test. Normally distributed data were summarized as mean ± standard deviation (SD) and compared using the independent-samples t-test, whereas non-normally distributed variables were reported as median (interquartile range [IQR]) and compared using the Mann–Whitney U test. Categorical variables were expressed as frequencies and percentages and compared using the chi-square or Fisher’s exact test, as appropriate.

Univariate logistic regression was performed to identify factors associated with 30-day mortality and clinical success. Variables with p < 0.10 in univariate analyses, along with pre-specified clinically essential covariates (age, Charlson Comorbidity Index, and sepsis or shock), were entered into multivariate logistic regression models using the enter method to determine independent predictors, with adjusted odds ratios (aORs) and 95% confidence intervals (CIs) reported. Survival analysis was conducted using the Kaplan–Meier method, and group differences were assessed with the log-rank test. A two-tailed *p*-value < 0.05 was considered statistically significant. A *post-hoc* analysis was performed to compare clinical and microbiological outcomes between standard-dose (100 mg daily) and high-dose (200 mg daily) tigecycline groups using Fisher’s exact test.

A sensitivity analysis was conducted, excluding patients with bloodstream or urinary tract infections to account for potential confounding related to infection source and tigecycline pharmacokinetics. In addition, a subgroup analysis of the combination group when tigecycline was combined with meropenem *vs* other antibiotics for the treatment of gram-negative infections, including colistin. The treatment outcomes between those groups were compared.

Given the observational design of the study, treatment allocation was not randomized and reflected clinician judgment. No propensity score matching was performed due to the marked imbalance in baseline severity and pathogen distribution between groups. All statistical analyses were performed using IBM SPSS Statistics, version 29.0 (IBM Corp., Armonk, NY, USA) and visuals were generated using GraphPad Prism 10.6.1.

## 3 Results

### 3.1 Baseline characteristics

**Figure 1:**
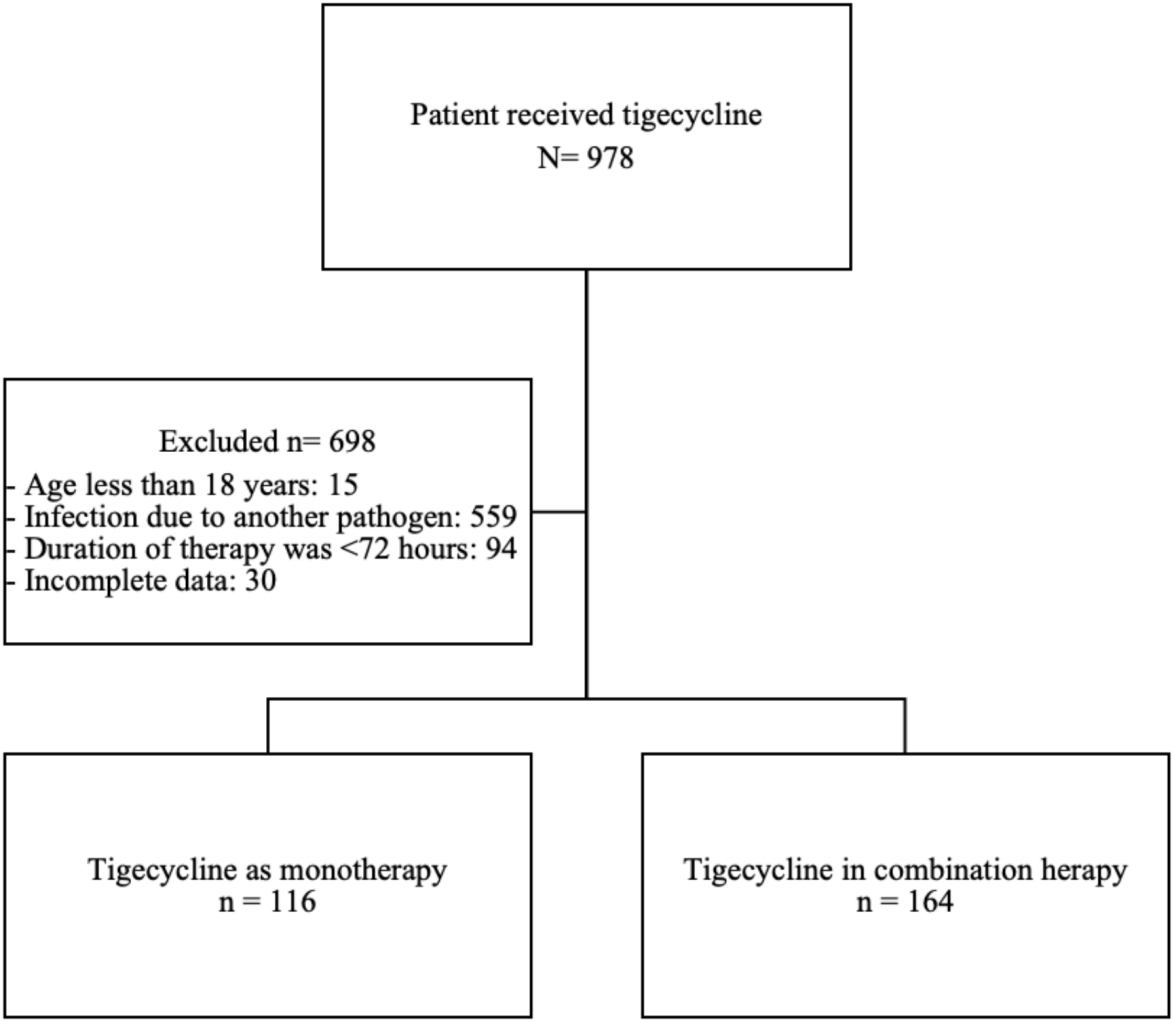
Flow chart of inclusion and exclusion.

We screened 978 patients who received tigecycline during the study period. Of these, 698 were excluded, primarily because 559 patients were treated for infections caused by bacteria outside the scope of this study (**Figure 1**). Ultimately, 280 patients met eligibility criteria and were included in the analysis, of whom 116 (41.4%) received tigecycline monotherapy and 164 (58.6%) received tigecycline in combination with another active antimicrobial agent.

**Table 1** presents the baseline characteristics. The mean age of the overall cohort was 59.5 ± 19.6 years, with no significant difference between the two groups (p = 0.809). Males comprised 64% of the study population. The most common comorbidities included diabetes mellitus (53.9%), chronic kidney disease (12.5%), and congestive heart failure (14.6%). Chronic kidney disease was significantly more prevalent among patients who received monotherapy (22.4% *vs*. 5.5%, *p* < 0.001), as was immunosuppressed status (14.7% *vs*. 7.3%, *p* = 0.047). The median Charlson Comorbidity Index (CCI) was 4 overall, with no significant difference between groups (4 *vs*. 5, *p* = 0.332).

**Table 1:** Baseline characteristics.

Variable	Total (n = 280)	Monotherapy (n =116)	Combination therapy (n = 164)	*P*-value
Demographics				
Age, mean ± SD (years)	59.5 ± 19.6	59.14 ± 18.57	59.71 ± 20.35	0.809
Male gender, n (%)	179 (64)	69 (59.6)	110 (67.1)	0.193
Weight, median (IQR), kg	73 (64-86.7)	76.7 (67.3-90)	71 (63-82.7)	0.667
BMI, mean ± SD	27.8 ± 9	28.32 ± 10.1	27.4 ± 8.24	0.417
Comorbidities, n (%)				
Myocardial infarction	32 (11.4)	12 (10.3)	20 (12.2)	0.632
Congestive heart failure	41 (14.6)	18 (15.5)	23 (14)	0.728
COPD	13 (4.6)	4 (3.4)	9 (5.5)	0.424
Liver disease	11 (3.9)	4 (3.4)	7 (4.3)	0.493
Diabetes mellitus	151 (53.9)	60 (51.7)	91 (55.5)	0.545
Chronic kidney disease	35 (12.5)	26 (22.4)	9 (5.5)	**<0.001**
Immunosuppressed	29 (10.4)	17 (14.7)	(12 (7.3)	**0.047**
CCI, median (IQR)	4 (1-6)	4 (3–6)	5 (2–6)	0.332
Clinical characteristics, n (%)				
Admission department, n (%) General ICU	194 (69.3) 86 (30.7)	92 (79.3) 24 (20.7)	102 (62.2) 62 (37.8)	**0.002**
ICU during admission	225 (80.4)	86 (74.1)	139 (84.8)	**0.028**
ICU duration, median (IQR), days	21 (12-36)	26 (12.5-37)	21 (15-35)	0.757
Sepsis or septic shock	131 (46.8)	51 (44)	80 (48.8)	0.426
Mechanical ventilation	194 (69.3)	71 (61.2)	123 (75)	**0.014**
Infection characteristics, n (%)				
Infection sources				
Urine	19(6.9)	10 (8.62)	9 (5.5)	0.340
Respiratory	127 (46.2)	45 (38.8)	82 (50)	0.068
Blood	38 (13.8)	15 (12.93)	24 (14.63)	0.729
Wound	69 (25.1)	32 (27.6)	38 (23.17)	0.4047
Other	22 (8)	14 (12.07)	11 (6.7)	0.139
Polymicrobial infection	108 (38.6)	41 (35.3)	67 (40.9)	0.351
Resistant isolate type				
ESBL (*E. coli* + *K. pneumoniae)*	37 (13.2)	18 (15.5)	19 (11.6)	0.339
CRE *K. pneumoniae*	104 (37.1)	73 (62.9)	31 (18.9)	**<0.001**
MDR *A. baumannii*	139 (49.6)	25 (21.6)	114 (69.5)	**<0.001**

Values are n (%) unless stated otherwise. Continuous data are reported as median (IQR) or mean ± SD, Bolded *p*-value indicate statistical significance.

IQR, interquartile range; BMI, body mass index; CKD, chronic kidney disease; COPD, chronic obstructive pulmonary disease; CVD, cardiovascular disease; DM, diabetes mellitus; ICU, intensive care unit; MI, myocardial infarction; PVD, peripheral vascular disease; ESBL, extended-spectrum β-lactamase; *E. coli*, *Escherichia coli*; *K. pneumoniae*, *Klebsiella pneumoniae*; CRE, Carbapenem-Resistant Enterobacterales; MDR, multidrug-resistant; *A. baumannii*, *Acinetobacter baumannii*; IQR, interquartile range; SD, standard deviation.

In terms of clinical characteristics, most patients were initially admitted to general wards (69.3%). Initial ICU admission was significantly more common among patients receiving combination therapy compared to monotherapy (37.8% *vs*. 20.7%, *p* = 0.002). Similarly, ICU admission at any point during hospitalization was higher in the combination therapy group (84.8% *vs*. 74.1%, *p* = 0.028), and mechanical ventilation was significantly more frequent (75% *vs*. 61.2%, *p* = 0.014).

Regarding infection sources, respiratory infections were the most common (46.2%), followed by wound infections (25.1%) and bloodstream infections (13.8%). The majority of isolates were MDR *A. baumannii* (49.6%), which was significantly more common among those who received combination therapy (69.5% *vs*. 21.6%, *p* < 0.001). In contrast, CRE *K. pneumoniae* was more frequent in the monotherapy group (62.9% *vs*. 18.9%, *p* < 0.001).

### 3.2 Tigecycline treatment characteristics

Most patients (94.6%) received a loading dose of tigecycline, with no significant difference between groups. Standard maintenance dosing of 100 mg daily was used in 90.7% of cases. In comparison, a high-dose maintenance dosing (200 mg daily) and adjusted-dose (50 mg daily for hepatic impairment) were used less frequently and did not differ significantly between groups. A *post-hoc* analysis comparing outcomes by dose group revealed no significant differences in clinical success (37.5% *vs*. 56.3%, p=0.209), 30-day mortality (37.5% *vs*. 25.2%, p=0.138), antibiotic escalation (37.5% *vs*. 29.9%, p=0.572), or microbiologic failure (40.0% *vs*. 41.0%, p=1.000) between high-dose and standard-dose groups. However, overall mortality was significantly higher in the high-dose group (68.8% *vs*. 41.7%, p=0.035) (**Supplementary Table 3**).

The median duration of therapy was 10 days (IQR 5–15.5) and did not differ significantly between monotherapy and combination therapy groups (*p* = 0.352). An early tigecycline start (< 48 h after culture) occurred in 58.2% of cases, with similar timing across both groups. The median time to tigecycline initiation was approximately 3 days (IQR 1–5).

Among patients receiving combination therapy, the most frequently co-administered agents were meropenem (44.5%), colistin (39%), and gentamicin (17.1%) (**Table 2**).

**Table 2:** Tigecycline treatment characteristics.

Variable	Total (n =280)	Monotherapy (n = 116)	Combination therapy (n = 164)	*P*-value
Dosing characteristics				
Received a loading dose, n (%)	265 (94.6)	108 (93.1)	157 (95.7)	0.336
Maintenance dose, n (%)				
100 mg daily dose	254 (90.7)	109 (94)	145 (88.4)	0.115
200 mg daily dose	16 (5.7)	5 (4.3)	11 (6.7)	0.395
50 mg (Dose adjusted)	12 (4.3)	4 (3.4)	8 (4.9)	0.395
Duration of therapy, median (IQR), days	10 (6-15.2)	10 (5-15.5)	9 (5-14)	0.352
Timing of therapy				
Early tigecycline start (<48h after culture), n (%)	163 (58.2)	67 (57.8)	96 (58.5)	0.897
Time to tigecycline start, median (IQR), days	3 (1-5)	3 (1-5)	3 (2-5)	0.804
Combination regimen				
Meropenem	—	—	73 (44.5)	—
Colistin	—	—	64 (39)	—
Gentamicin	—	—	28 (17.1)	—
Piperacillin/Tazobactam	—	—	16 (9.8)	—
Amikacin	—	—	8 (4.9)	—
Ciprofloxacin	—	—	6 (3.7)	—
Imipenem	—	—	9 (3.2)	—
Trimethoprim/sulfamethoxazole	—	—	4 (2.8)	—
Ceftazidime/Avibactam	—	—	3 (1.1)	—

Values are n (%) unless stated otherwise. Continuous data are reported as median (IQR) or mean ± SD, Bolded *p*-value indicate statistical significance.

IQR, interquartile range.

### 3.3 Clinical and microbiological outcomes

Overall clinical success was achieved in 53.2% of patients. Success rates were significantly higher with monotherapy compared to combination therapy (64.7% *vs*. 45.1%, *p* = 0.001). Treatment escalation during tigecycline therapy was more common among those on combination regimens (36.6% *vs*. 19%, *p* = 0.001). Among the 131 patients who did not achieve clinical success, 49 (37.4%) experienced 30-day mortality without antibiotic escalation, 58 (44.3%) required antibiotic escalation, and 24 (18.3%) experienced both antibiotic escalation and 30-day mortality. In terms of microbiologic outcomes, repeated culture collection within 21 days was more common among patients receiving combination therapy (67.1% *vs*. 52.6%, p = 0.014). However, the rate of microbiological failure did not differ significantly between the two treatment groups (42.6% vs. 40.0%, p = 0.738). Treatment escalation during tigecycline therapy was more common among those on combination regimens (36.6% *vs*. 19%, *p* = 0.001). (**Table 3**).

**Table 3:** Clinical and microbiologic outcomes.

Variable	Total (n =280)	Monotherapy (n =116)	Combination therapy (n =164)	*P*-value
Clinical outcomes				
Clinical success, n (%)	149 (53.2)	75 (64.7)	74 (45.1)	*0.001
Escalation during tigecycline, n (%)	82 (29.3)	22 (19)	60 (36.6)	*0.001
30-day mortality, n (%)	73 (26.1)	26 (22.4)	47 (28.7)	0.241
Overall mortality, n (%)	117 (41.8)	44 (37.9)	73 (44.5)	0.271
Microbiologic outcomes				
Microbiologic failure, n (%)	70 (40.9)	26 (42.6)	44 (40)	0.738
Repeated culture within 21 days, n (%)	171 (61.1)	61 (52.6)	110 (67.1)	*0.014
Same pathogen regrowth, n (%)	72 (42.1)	26 (42.6)	46 (41.8)	0.919
Change in susceptibility to tigecycline, n (%)	5 (7.6)	0	5 (12.5)	0.074
Other outcomes				
Length of stay, median (IQR), days	48.5 (27-91.5)	65 (34 -105)	48 (24-74)	0.102
Time from culture to outcome, median (IQR), days	35 (17-82.5)	24 (14-44)	23 (14-38)	0.780
Time from tigecycline to outcome, median (IQR), days	28 (12-77.5)	18 (7-36.5)	19 (9-37)	0.418
ID consultation, n (%)	225 (80.4)	95 (82)	130 (79.3)	0.586

Values are n (%) unless stated otherwise. Continuous data are reported as median (IQR) or mean ± SD, Bolded *p*-value indicate statistical significance.

IQR, interquartile range; ID. infectious diseases.

Thirty-day mortality was 26.1% overall, with no significant difference between groups. Similarly, in-hospital mortality did not differ significantly between groups (37.9% *vs*. 44.5%, *p* = 0.271). The median hospital length of stay was 48.5 days (IQR 27–91.5), with no significant difference between treatment groups. Similarly, the time from culture to outcome and from tigecycline initiation to outcome were comparable. Infectious disease consultation was obtained in 80.4% of cases overall, without significant differences between treatment groups (**Table 3**).

In a sensitivity analysis excluding patients with bloodstream and urinary tract infections, clinical success did not differ significantly between groups (37% *vs*. 34%, *p* = 0.070). Treatment escalation during tigecycline therapy remained significantly more frequent among patients receiving combination therapy (37.0% *vs*. 19.0%, p = 0.004). Similarly, repeated culture collection within 21 days was more common in the combination therapy group (p = 0.017). 30-day mortality, overall mortality, microbiologic failure, and same-pathogen regrowth rates remained comparable between the two groups (**Table 4**).

**Table 4:** Analysis of clinical and microbiologic outcomes, excluding blood and urine sources of infection.

Variable	Total (n =223)	Monotherapy (n =91)	Combination therapy (n =131)	*P*-value
Clinical outcomes				
Clinical success, n (%)	68 (30.5)	34 (37)	34 (26)	0.070
Escalation during tigecycline, n (%)	65 (29.1)	17 (19)	48 (37)	*0.004
30-day mortality, n (%)	107 (48)	42 (46)	65 (50)	0.6
Overall mortality, n (%)	86 (38.5)	29 (32)	57 (44)	0.080
Microbiologic outcomes				
Microbiologic failure, n (%)	67 (30)	25 (58)	42 (51)	0.4
Repeated culture within 21 days, n (%)	126 (56.5)	43 (47)	83 (63)	*0.017
Same pathogen regrowth, n (%)	126 (56.5)	25 (58)	44 (53)	0.6
Change in susceptibility to tigecycline, n (%)	4 (1.8)	0 (0)	4 (11)	0.15
Other outcomes				
Length of stay, median (IQR), days	49 (34-84)	70 (45-118)	48 (27-68)	*0.003
Time from culture to outcome, median (IQR), days	37 (18-84)	41 (21-86)	36 (17-83)	0.4
Time from tigecycline to outcome, median (IQR), days	34 (15-81)	34 (17-82)	36 (17-83)	0.6
ID consultation, n (%)	108(48.4)	73 (80)	35 (15-78)	0.6

Values are n (%) unless stated otherwise. Continuous data are reported as median (IQR) or mean ± SD. Bolded *p*-value indicates statistical significance.

IQR: interquartile range; ID: infectious diseases.

**Figure 2:**
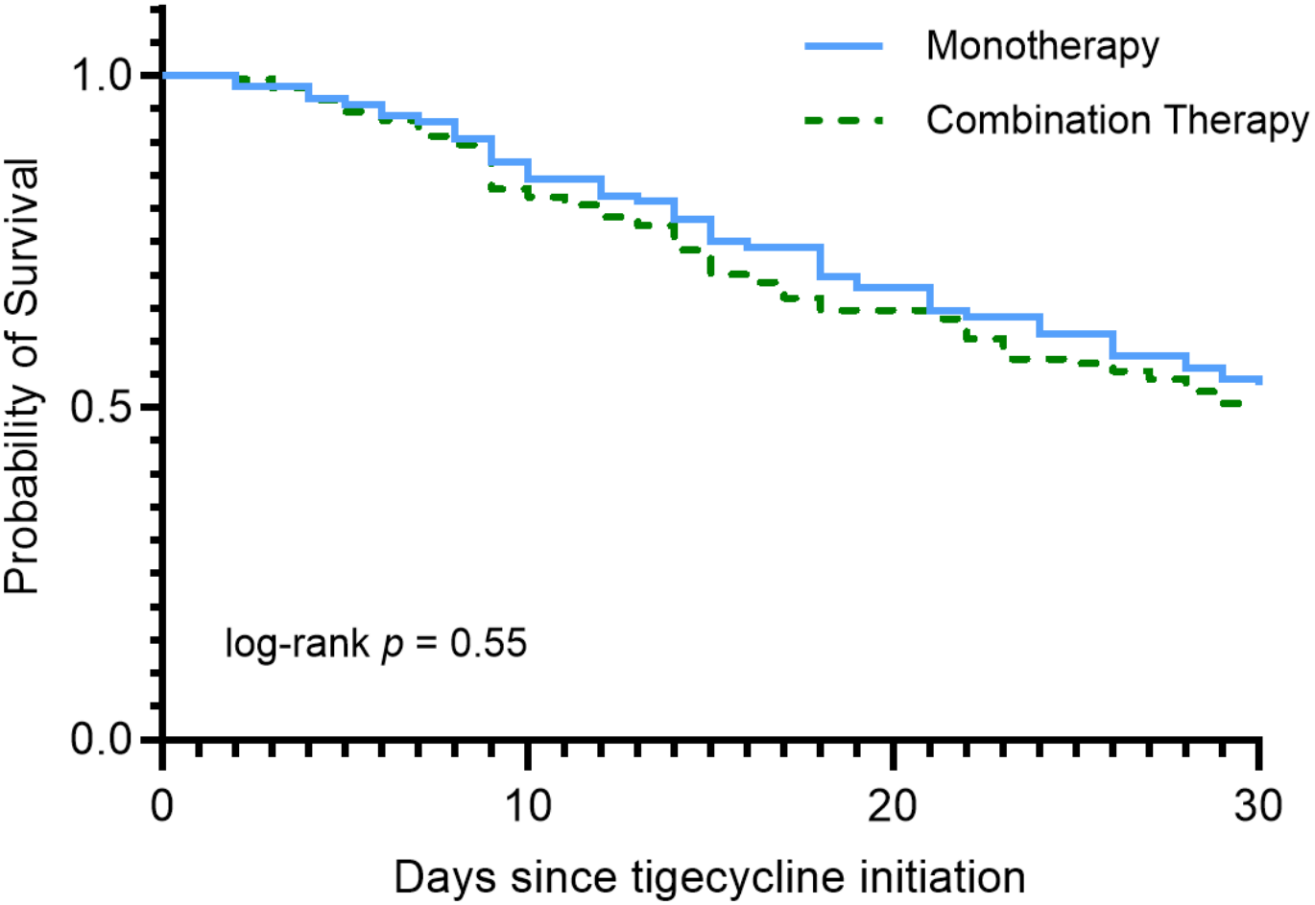
Kaplan–Meier 30-day survival following tigecycline initiation: monotherapy *vs*. combination therapy. Kaplan–Meier survival analysis showing 30-day survival following tigecycline initiation in patients treated with monotherapy (blue solid line) or combination therapy (green dashed line). Survival probabilities were not significantly different between groups (log-rank p = 0.55; Wilcoxon p = 0.48; HR = 0.90, 95% CI 0.64–1.27).

Kaplan–Meier survival analysis demonstrated no significant difference in 30-day survival between monotherapy and combination therapy (**Figure 2**). The log-rank test showed no significant difference between groups (χ² = 0.36, p = 0.55), and the hazard ratio for mortality with combination therapy compared to monotherapy was 0.90 (95% CI, 0.64–1.27). Median survival was not reached in either group, as more than half of patients survived beyond 30 days.

### 3.4 Univariate and multivariate logistic regression

In univariate analyses (**Supplementary Table 1**), none of the evaluated clinical, microbiologic, or treatment-related variables were significantly associated with 30-day mortality following tigecycline initiation. Early tigecycline start demonstrated a nonsignificant trend toward lower mortality (OR 0.64; 95% CI, 0.40–1.03; p = 0.066), but no other covariates approached the predefined threshold for inclusion based solely on univariate significance. In The multivariable model, which incorporated early tigecycline start along with clinically essential covariates (age, comorbidity burden, and presence of sepsis or shock), did not identify any independent predictors of 30-day mortality. Early tigecycline start continued to show a nonsignificant protective association (aOR 0.64; 95% CI, 0.38– 1.06; p = 0.085), while age (aOR 1.01; 95% CI, 0.99–1.03; p = 0.559), Charlson Comorbidity Index (aOR 1.02; 95% CI, 0.89–1.16; p = 0.824), and sepsis or shock (aOR 0.94; 95% CI, 0.57–1.56; p = 0.814) were not associated with mortality in the adjusted model.

Regarding clinical success (**Supplementary Table 2**), univariate analysis showed that sepsis or shock, ICU admission, and combination therapy were significantly associated with lower odds of clinical success. After multivariable adjustment in the logistic regression model, none of these variables remained statistically significant, although combination therapy (AOR 0.59, 95% CI 0.31–1.13; *p* = 0.113) and microbiologic failure (AOR 1.69, 95% CI 0.89–3.20; *p* = 0.108) showed nonsignificant trends.

## 4 Discussion

In this real-world cohort study of patients treated with tigecycline for MDR gram-negative infections, we observed several key findings. The cohort was characterized by a high burden of severe illness and the predominant pathogens were CRE *Klebsiella pneumoniae* and MDR *Acinetobacter baumannii*, with MDR *A. baumannii* being significantly more frequent among patients who received combination therapy. Tigecycline was administered in combination with other antimicrobials in nearly 60% of cases, particularly in critically ill patients, reflecting real-world practice in a high-resistance setting with limited therapeutic options. The most common combination was with meropenem, colistin, and gentamicin. Although unadjusted analyses demonstrated higher clinical success among patients receiving tigecycline monotherapy, no significant differences were observed in mortality or microbiological outcomes between monotherapy and combination therapy after accounting for confounding factors.

The apparent advantage of monotherapy in unadjusted analysis is likely attributable to differences in patient characteristics and illness severity rather than a true therapeutic superiority of tigecycline alone. Patients receiving combination therapy had a higher proportion of ICU admissions, mechanical ventilation, and infections predominantly due to MDR *A. baumannii*, which may have contributed to worse outcomes despite broader antimicrobial coverage. Conversely, monotherapy recipients were more likely to have infections caused by CRE *K. pneumoniae* and fewer markers of critical illness. These baseline imbalances strongly suggest that clinicians reserved combination therapy for patients with advanced disease or limited expected response, thereby biasing unadjusted outcome comparisons. The significant imbalance in pathogen distribution between treatment groups, where combination therapy was used in 69.5% of *A. baumannii* cases and monotherapy in 62.9% of CRE *K. pneumoniae* cases, further underscores the role of pathogen-specific factors in influencing clinical outcomes. This distribution likely reflects the clinical perception of *A. baumannii* as an inherently more ‘difficult-to-treat’ pathogen, given its capacity to develop pan-resistance through diverse mechanisms, including efflux pumps and beta-lactamase production. In our cohort, *A. baumannii* was isolated more frequently from patients with higher illness severity markers, such as those requiring mechanical ventilation. These inherent characteristics may have independently contributed to poorer outcomes in the combination group, a phenomenon consistent with prior research by Lee et al. (2013), which suggests that clinical success in such infections is often driven more by host factors and disease severity than the specific antibiotic regimen. Consequently, the higher utilization of combination therapy for *A. baumannii* likely serves as a proxy for infection complexity rather than an indicator of therapeutic efficacy (Lee et al., 2013).

Importantly, the disappearance of the monotherapy advantage after multivariate adjustment supports the interpretation that regimen selection primarily served as a marker of illness severity rather than a determinant of efficacy. Importantly, our sensitivity analysis excluding patients with bloodstream and urinary tract infections provides additional insight into the robustness of these findings. The clinical success remained numerically higher with monotherapy but did not reach statistical significance, further supporting the notion that the observed differences in the overall cohort were driven by confounding by indication. However, treatment escalation during tigecycline therapy and repeated culture collection within 21 days remained significantly more frequent among patients receiving combination therapy. These findings likely reflect persistent clinical instability or lack of early response rather than inferior antimicrobial activity, as microbiologic failure and same-pathogen regrowth rates remained comparable between groups. Collectively, these results reinforce that combination therapy often served as a marker of disease severity and therapeutic uncertainty rather than a strategy that independently improved outcomes.

In our study, none of the evaluated clinical, microbiologic, or treatment-related variables were significantly associated with 30-day mortality in univariate analyses, although early tigecycline initiation demonstrated a nonsignificant trend toward lower mortality. Importantly, these findings persisted after multivariable adjustment, suggesting that mortality in this severely ill population was driven more by global disease severity and host factors not fully captured by available covariates than by specific antimicrobial strategies.

In contrast, several markers of illness severity such as sepsis, ICU admission during hospitalization, and receipt of combination therapy, were significantly associated with lower odds of clinical success in univariate analysis. However, these did not remain significant after controlling for confounders. The absence of a significant association between combination therapy and clinical success after adjustment aligns with prior literature, where combination therapy often reflected greater baseline disease severity rather than improved efficacy. The lack of an independent association between combination therapy and clinical success after adjustment aligns with prior literature in which combination regimens were preferentially used in more critically ill patients without consistently improving outcomes. For example, while a systematic review by Wang et al. (2017) of tigecycline for bloodstream infections reported a statistically significant increase in clinical cure compared to control regimens, they also revealed a concerning safety signal where monotherapy was associated with a higher mortality risk compared with combination therapy for bloodstream infections (Wang et al., 2017). Conversely, a more recent propensity score-matched study by Zha et al. (2023) on nosocomial pneumonia caused by carbapenem-resistant *K. pneumoniae* and *A. baumannii* found that adding Polymyxin B to high-dose tigecycline offered no significant benefit over monotherapy in terms of 14-day mortality or clinical cure, ultimately advocating against its routine adjunctive use (Zha et al., 2023). Collectively, these findings underscore the need for cautious interpretation of monotherapy versus combination outcomes, recognizing the overriding influence of underlying disease severity and the varied efficacy of specific combination partners across different infection sites.

Although high-dose tigecycline (200 mg daily) is often recommended to optimize the AUC/MIC ratio in severe infections caused by MDR pathogens, only 16 patients (5.7%) in our cohort received this regimen. Standard-dose tigecycline is well recognized to achieve suboptimal serum concentrations, and dose escalation has been proposed as a pharmacokinetic strategy to improve target attainment, particularly against pathogens with elevated MICs (Barbour et al., 2009; De Pascale et al., 2020). A systematic review and meta-analysis by Zha et al. demonstrated that high-dose tigecycline was associated with lower all-cause mortality and higher clinical cure rates compared to standard dosing (Zha et al., 2020). Similarly, Bosso et al. reported that high-dose therapy was the sole independent predictor of clinical cure in ventilator-associated pneumonia caused by MDR Gram-negative bacteria (De Pascale et al., 2014). In our cohort, no significant differences were observed in clinical and microbiological between dose groups. However, overall mortality was significantly higher in the high-dose group (68.8% *vs*. 41.7%, p=0.035), suggesting that clinicians preferentially prescribed higher doses in more critically ill patients, introducing channeling bias that precludes meaningful dose-outcome comparisons. This observation is consistent with prior reports noting that high-dose recipients are often sicker at baseline (Falagas et al., 2014). The small size of the high-dose group (n=16) further limits statistical power. The absence of MIC data in our study is an additional limitation, as high-dose therapy is most likely to benefit patients with isolates at the upper end of the MIC distribution (De Pascale et al., 2020; Zha et al., 2020).

In our cohort, respiratory infections accounted for nearly half of all cases, a factor that likely influenced both treatment selection and outcomes. The substantial proportion of critically ill patients with pneumonia may also explain the relatively high overall mortality observed, despite aggressive antimicrobial therapy. These considerations reinforce guideline recommendations discouraging tigecycline monotherapy for bloodstream infections and severe hospital-acquired pneumonia (Kalil et al., 2016; Park et al., 2024). Pharmacokinetic and pharmacodynamic considerations further contextualize our findings. Tigecycline exhibits a high volume of distribution with low peak serum concentrations, making attainment of optimal AUC/MIC ratios challenging in critically ill patients (Yaghoubi et al., 2022). Although high-dose tigecycline regimens have been proposed as a strategy to overcome these limitations, our study did not demonstrate a statistically significant benefit with a 200 mg daily dose after adjustment (De Pascale et al., 2014). However, this finding should be interpreted with caution, as high-dose therapy was used in a small subset of severely ill patients, resulting in wide confidence intervals and limited statistical power. Importantly, the absence of a detected benefit does not equate to evidence of inefficacy; rather, it underscores the difficulty of achieving reliable exposure in advanced infection with dose escalation alone.

An important factor influencing tigecycline efficacy is the MIC of the infecting pathogen. Tigecycline activity is best described by the pharmacokinetic/pharmacodynamic index of AUC/MIC, and higher MIC values may significantly reduce the probability of target attainment in critically ill patients. Clinical breakpoints for tigecycline are currently limited; the Clinical and Laboratory Standards Institute provides susceptibility breakpoints only for Enterobacterales (susceptible ≤2 mg/L), while no validated breakpoints exist for *Acinetobacter baumannii*. Furthermore, the European Committee on Antimicrobial Susceptibility Testing has removed clinical breakpoints for tigecycline against several Gram-negative pathogens due to concerns regarding pharmacokinetic target attainment (Clinical and Laboratory Standards Institute, 2024; European Committee on Antimicrobial Susceptibility Testing, 2024).

Our study has several limitations. The retrospective, single-center design limits causal inference and generalizability, and introduces an inherent risk of confounding, despite rigorous adjustment for measured confounders. Although ICU admission, mechanical ventilation, sepsis, and the Charlson Comorbidity Index were used as proxies for illness severity and included in multivariable regression analysis adjusted for ICU admission, sepsis or shock, and Charlson Comorbidity Index as covariates, validated ICU severity scores (e.g., APACHE II) were not uniformly available. Therefore, residual confounding due to unmeasured severity factors cannot be entirely excluded. In addition, combination therapy encompasses heterogeneous agents with differing pharmacodynamic profiles, potentially masking regimen-specific effects. Moreover, detailed MIC distributions for individual isolates were not consistently available in the microbiology database; therefore, we were unable to evaluate the relationship between tigecycline MIC values and clinical outcomes. Variability in pathogen MICs may partially explain the observed clinical success rate and represents an important consideration when interpreting tigecycline effectiveness in real-world settings. Finally, the relatively small sample size for specific pathogen-site combinations limited the power of our subgroup analyses.

In conclusion, this real-world study demonstrates that tigecycline is frequently used for MDR gram-negative infections in severely ill patients, often as part of combination regimens. After adjustment and sensitivity analysis excluding bloodstream and urinary tract infections, neither monotherapy nor combination therapy conferred a clear outcome advantage. These findings emphasize that patient condition, infection site, overall severity of illness, and timely supportive care remain the primary drivers of outcome. Tigecycline continues to have a role as a salvage agent; however, its use should be individualized, with careful consideration of pharmacokinetic limitations and the specific clinical context. Future prospective studies with infection-site stratification, larger sample size per pathogen, and standardized severity scoring systems should evaluate specific combination regimens to reduce heterogeneity and provide more definitive comparative evidence regarding the optimal role of tigecycline in the management of MDR gram-negative infections.

## Data Availability

The data analyzed in this study is subject to the following licenses/restrictions: The datasets generated during and/or analyzed during the current study are available from the corresponding author on reasonable request. Requests to access these datasets should be directed to owaiss@ksau-hs.edu.sa.
