# Supplementary material for: Characteristics and outcomes of patients treated with tigecycline for MDR gram-negative infections: a retrospective cohort study
Source: Front Cell Infect Microbiol. 2026 Apr 1;16:1790441. doi: 10.3389/fcimb.2026.1790441 (PMC13079584; doi:10.3389/fcimb.2026.1790441)
Supplement: Supplementary file 1 [file Table1.docx]

**Table S1. Predictors of 30-day Mortality on Tigecycline Therapy (Univariate and Multivariate Logistic Regression)**

| Variable | OR (95% CI) | *p*-value | AOR (95% CI) | *p*-value |
| --- | --- | --- | --- | --- |
| Age (per year) | 1.01 (0.99 – 1.02) | 0.131 | 1.01 (0.99 – 1.03) | 0.559 |
| Charlson Comorbidity Index | 1.05 (0.97 – 1.15) | 0.242 | 1.02 (0.89 – 1.16) | 0.824 |
| Polymicrobial infection | 1.06 (0.65 – 1.71) | 0.820 | — | — |
| Sepsis or shock | 0.94 (0.59 – 1.50) | 0.781 | 0.94 (0.57 – 1.56) | 0.814 |
| Mechanical ventilation | 0.84 (0.5-1.4) | 0.511 |  |  |
| Tigecycline 200 mg dose | 0.63 (0.22 – 1.78) | 0.381 | — | — |
| Early tigecycline start | 0.64 (0.40 – 1.03) | 0.066 | 0.64 (0.38 – 1.06) | 0.085 |
| Meropenem/Imipenem | 1.19 (0.71 – 1.98) | 0.517 | — | — |
| Colistin | 1.19 (0.68 – 2.08) | 0.542 | — | — |
| Respiratory culture source | 0.72 (0.45 – 1.15) | 0.166 | — | — |
| Blood culture source | 0.98 (0.50 – 1.92) | 0.946 | — | — |
| *A. baumannii* | 1.33 (0.83 – 2.13) | 0.234 | — | — |
| Combination therapy | 1.12 (0.70 – 1.80) | 0.640 | — | — |
| Microbiologic failure | 0.87 (0.48 – 1.61) | 0.666 | — | — |
