# Supplementary material for: Characteristics and outcomes of patients treated with tigecycline for MDR gram-negative infections: a retrospective cohort study
Source: Front Cell Infect Microbiol. 2026 Apr 1;16:1790441. doi: 10.3389/fcimb.2026.1790441 (PMC13079584; doi:10.3389/fcimb.2026.1790441)
Supplement: Supplementary file 2 [file Table2.docx]

**Table S2. Predictors of Clinical Success on Tigecycline Therapy (Univariate and Multivariate Logistic Regression)**

| Variable | OR (95% CI) | *p*-value | AOR (95% CI) | *p*-value |
| --- | --- | --- | --- | --- |
| Age (per year increase) | 0.99 (0.98–1.00) | 0.155 | — | — |
| Charlson Comorbidity Index | 0.98 (0.90–1.07) | 0.694 | — | — |
| Polymicrobial infection | 0.86 (0.53–1.40) | 0.543 | — | — |
| Sepsis or shock | 0.57 (0.35–0.92) | **0.020** | 1.00 (0.51–1.99) | 0.991 |
| ICU during admission | 0.44 (0.23–0.82) | **0.010** | 0.66 (0.23–1.87) | 0.662 |
| Tigecycline 100 mg dose | 1.62 (0.72–3.67) | 0.245 | — | — |
| Tigecycline 200 mg dose | 0.51 (0.18–1.44) | 0.202 | — | — |
| Early tigecycline start (<48 h) | 0.63 (0.39–1.02) | 0.061 | — | — |
| Combination therapy (vs monotherapy) | 0.45 (0.28–0.73) | **0.001** | 0.59 (0.31–1.13) | 0.113 |
| Microbiologic failure | 1.73 (0.93–3.20) | 0.082 | 1.69 (0.89–3.20) | 0.108 |

**Footnote:** Bolded p-value indicates statistical significance.

**Abbreviations:** ICU, intensive care unit
